# Supplementary figures and images for: Short-Chain Fructooligosaccharide Synthesis from Sugarcane Syrup with Commercial Enzyme Preparations and Some Physical and Antioxidation Properties of the Syrup and Syrup Powder
Source: Foods. 2023 Jul 29;12(15):2895. doi: 10.3390/foods12152895 (PMC10417662; doi:10.3390/foods12152895)

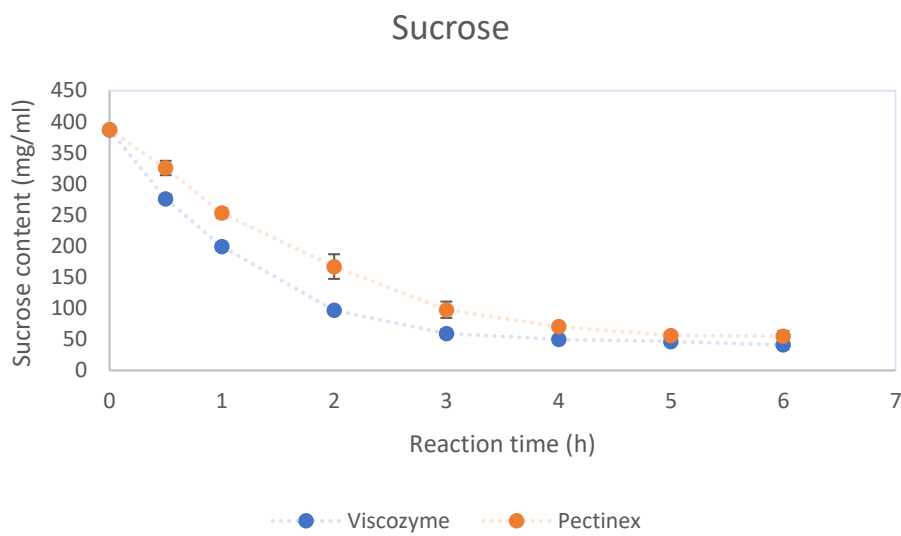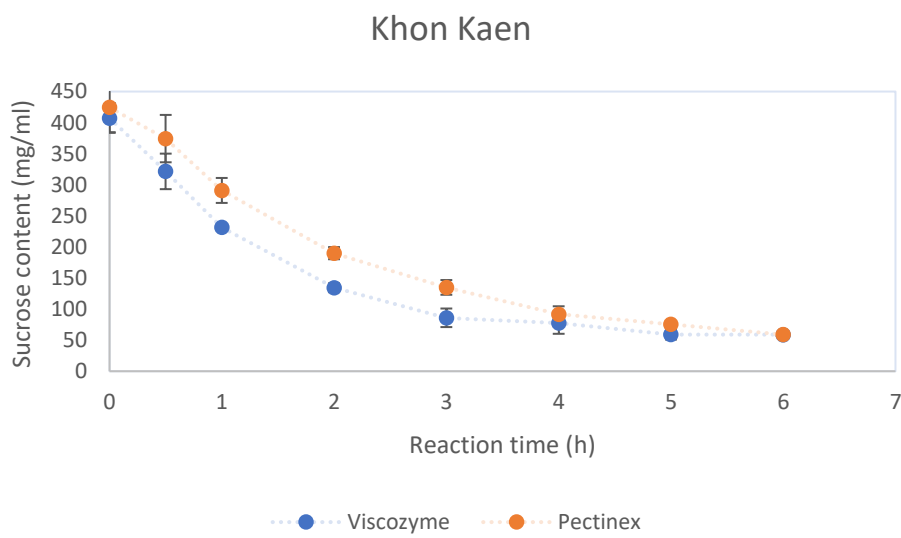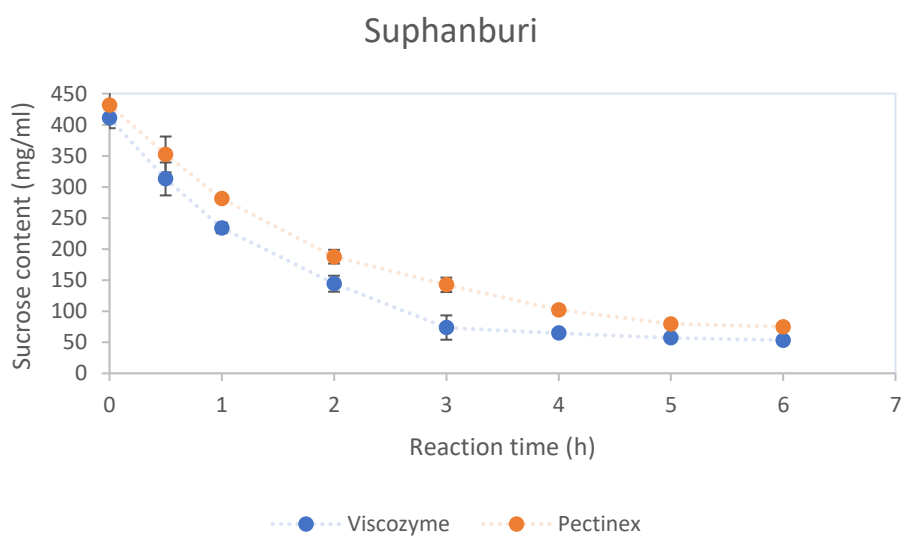

Figure S1. Changes in sucrose concentration during sc-FOS synthesis.

Supplement: Supplementary file 1 [file foods-12-02895-s001.zip › foods-2487685-supplementary.pdf]
